# Inactivation of ribosomal protein S27-like confers radiosensitivity via the Mdm2-p53 and Mdm2–MRN–ATM axes

**DOI:** 10.1038/s41419-017-0192-3

**Published:** 2018-02-02

**Authors:** Yongchao Zhao, Mingjia Tan, Xia Liu, Xiufang Xiong, Yi Sun

**Affiliations:** 1 Institute of Translational Medicine, Zhejiang University School of Medicine, Hangzhou, China; 2 Division of Radiation and Cancer Biology, Department of Radiation Oncology, University of Michigan, Ann Arbor, MI USA; 3 Key Laboratory of Combined Multi-Organ Transplantation, Ministry of Public Health, First Affiliated Hospital, Zhejiang University School of Medicine, Hangzhou, China; 4 Collaborative Innovation Center for Diagnosis and Treatment of Infectious Diseases, Zhejiang University, Hangzhou, China

## Abstract

RPS27L (ribosomal protein S27-like) is an evolutionarily conserved ribosomal protein and a direct p53 target. We recently reported that *Rps27l* disruption triggers ribosomal stress to induce p53, causing postnatal death, which can be rescued by *Trp53*^*+/−*^. Whether and how Rps27l modulates radiosensitivity is unknown. Here we report that *Rps27l*^*−/−*^*; Trp53*^*+/−*^ mice are extremely sensitive to radiation due to reduced proliferation and massive induction of apoptosis in radiation-sensitive organs. Mechanistically, the radiation sensitivity is mediated by two signaling pathways: (1) activated p53 pathway due to imbalanced Mdm2/Mdm4 levels and reduced E3 ligase activity; and (2) reduced DNA damage response due to reduced MRN/Atm signal as a result of elevated Mdm2 binding of Nbs1 to inhibit Nbs1–Atm binding and subsequent Atm activation. Indeed, heterozygous deletion of *Mdm2* restores the MRN/Atm signal. Collectively, our study revealed a physiological condition under which Rps27l regulates the Mdm2/p53 and MRN/Atm axes to maintain DNA damage response and to confer radioprotection in vivo.

## Introduction

The genome of a cell is constantly damaged by internal metabolites and environmental insults, such as ultraviolet light, ionizing radiation (IR), drug exposure, and oxidative stress^1^. To ensure maintenance of genomic stability, cells have evolved DNA damage response (DDR), a global signaling network to sense DNA damage and trigger distinct cellular responses including DNA repair, cell cycle arrest, senescence, and apoptosis^2,3^.

Specifically, upon DNA damage with DSBs (double-stranded breaks), Mre11-Rad50-Nbs1 (MRN) complex acts as a sensor to recruit ATM to the damage sites by directly binding of ATM with C terminus of Nbs1^4–6^. ATM is subsequently activated via autophosphorylation, which in turn phosphorylates its downstream effectors, including all three members of MRN complex, Chk2, H2AX, p53, and BRCA1 among others, to mediate distinct downstream responses^7^. In contrast, ATR is activated by damage-induced Replication Protein A (RPA)-coated single-stranded DNA and DNA replication stress to directly phosphorylate and activate Chk1 and other substrates. As a result, activated ATM–Chk2 and ATR–Chk1 pathways maintain genomic stability by triggering multiple cellular responses^8,9^.

Ribosomal proteins (RPs) are not only the essential components of the ribosome, but also play important roles in assembly of ribosome particles, a process termed as ribosome biogenesis^10^. Perturbation of ribosome biogenesis by multiple stresses, such as DNA damage, RP mutations, drug insults, nutrient deprivation, or oncogenic activation triggers nucleolar stress, also known as ribosomal stress (for review, see ref. 11). In response to ribosomal stress, a number of RPs were found to release from ribosome and directly bind with MDM2 to inhibit its ligase activity towards p53, leading to p53 activation, followed by p53-dependent cell cycle arrest and apoptosis (for reviews, see refs. 12–15). Given p53 acting as a guardian of the genome^16^, RPs could play significant roles in maintenance of genome integrity in a p53-dependent manner.

RPS27L (NM_015920) is an evolutionarily conserved ribosomal protein with 84-amino acid, which differs from its family member RPS27 (NM_001030) only by three amino acids (R5K, L12P, K17R). We and the others have previously reported that RPS27L is a direct p53 transcriptional target^17,18^. Our recent in vivo study showed that *Rps27l* disruption triggers ribosomal stress to stabilize Mdm2, which degrades Mdm4 to reduce Mdm2-Mdm4 E3 ligase activity towards p53, leading to p53-dependent apoptotic depletion of hematopoietic stem cells and postnatal death, which can be rescued by heterozygous deletion of *Trp53*^19^. *Rps27l* deletion also enhances lymphomagenesis in *Trp53*^*+/−*^ background by causing genomic instability to selectively lose *Trp53* heterozygosity^19^. However, whether and how Rps27l affects the efficiency of DNA damage response and modulates radiosensitivity in vivo is previously unknown.

Here we report that inactivation of Rps27l in a *Trp53*^*+/−*^ background significantly enhances the sensitivity of mice to ionizing radiation as a consequence of reduced proliferation and massive apoptosis in multiple radiation-sensitive organs. Involving mechanisms include (1) imbalanced levels of Mdm2 and Mdm4, leading to subsequent p53 activation and (2) elevated Mdm2 binding of Nbs1 to abrogate MRN complex for ATM activation, leading to reduced DNA damage response. Thus, Rps27l regulates radiation sensitivity in both p53-dependent and p53-independent manners and might serve as an attractive target for radiosensitization.

## Results

### *Rps27l* inactivation sensitizes *Trp53*^*+/−*^ mice to radiation

**Fig. 1:**
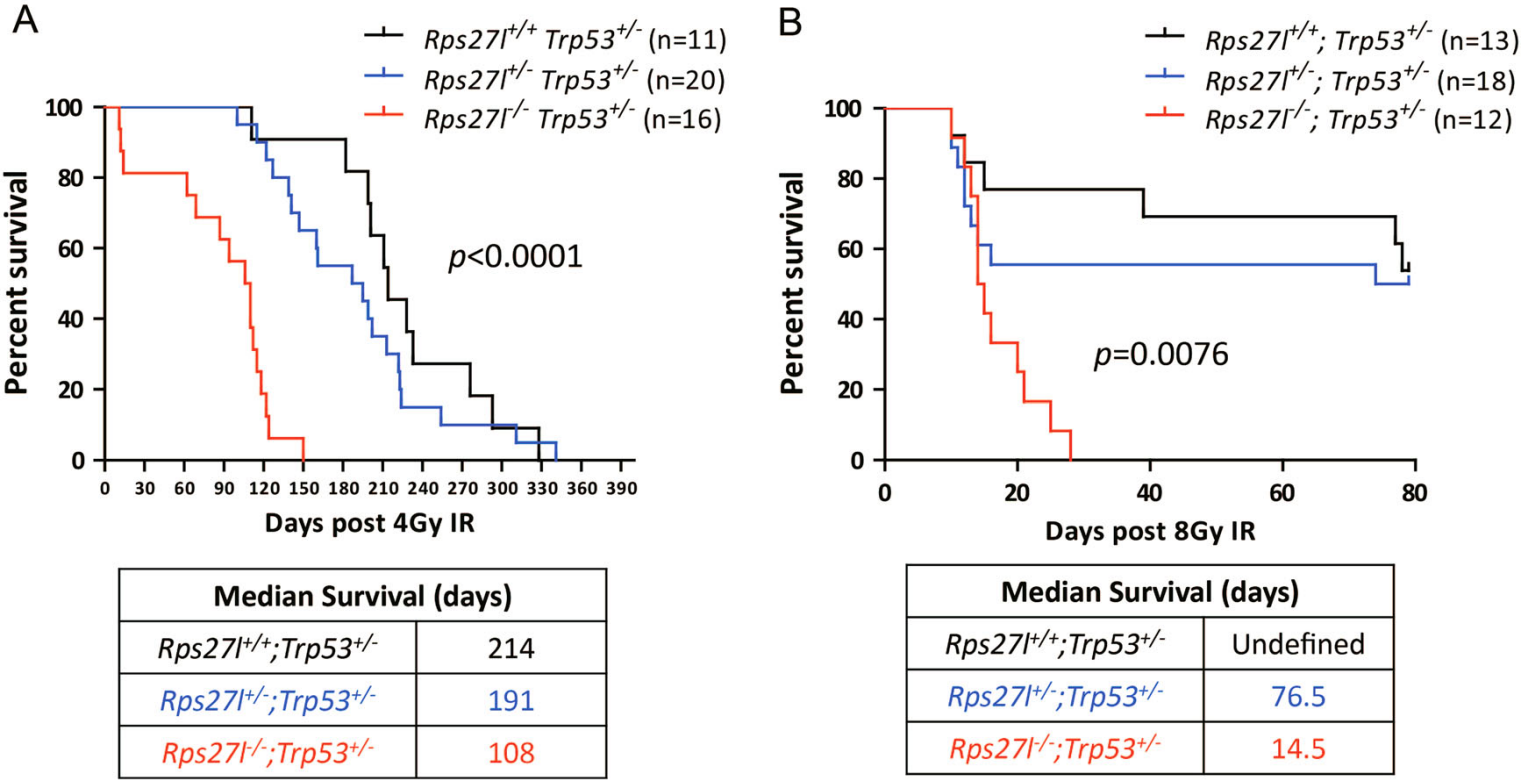
*Rps27l* inactivation sensitizes *Trp53*^*+/*−^ mice to radiation. Inactivation of *Rps27l* shortens the life span of *Trp53*^+/−^ mice after whole-body radiation. Three groups of mice at age of 5 weeks **a** or 8–10 weeks **b** with indicated genotypes were irradiated at 4 Gy (a) and 8 Gy (b), respectively, followed by survival monitor. Kaplan–Meier survival curves were shown. Log-rank test, statistical *p* values were shown

Our previous study showed that Rps27l regulates genomic stability^19^, whose disruption causes postnatal death, which can be rescued by heterozygous deletion of *Trp53*. The viability of *Rps27l*^*−/−*^*; Trp53*^*+/−*^ mice provided us an opportunity to study the role of Rps27l in radiation-induced tumorigenesis. We treated three genotypes of *Rp27l* mice, all under the *Trp53*^*+/−*^ background with whole-body radiation at the dose of 4 Gy. Unexpectedly, we found that *Rps27l*^*−/−*^; *Trp53*^*+/−*^ mice are very sensitive to radiation with a median survival of 108 days and all death at 150 days before tumor development. The difference is statistically significant (*p* < 0.0001), as compared to *Rps27l*^*+/+*^; *Trp53*^*+/−*^ or *Rps27l*^*+/−*^; *Trp53*^*+/−*^ mice, which have no statistical difference between each other (*p* > 0.05) (Fig. 1a). High radiosensitivity was further confirmed when *Rps27l*^*−/−*^; *Trp53*^*+/−*^ mice was exposed to 8 Gy radiation, which led to a median survival of 14.5 days and all death at 28 days. Again, the difference is statistically significant from *Rps27l*^*+/+*^*; Trp53*^*+/−*^ or *Rps27l*^*+/−*^; *Trp53*^*+/−*^ mice (*p* = 0.0076) (Fig. 1b).

**Fig. 2:**
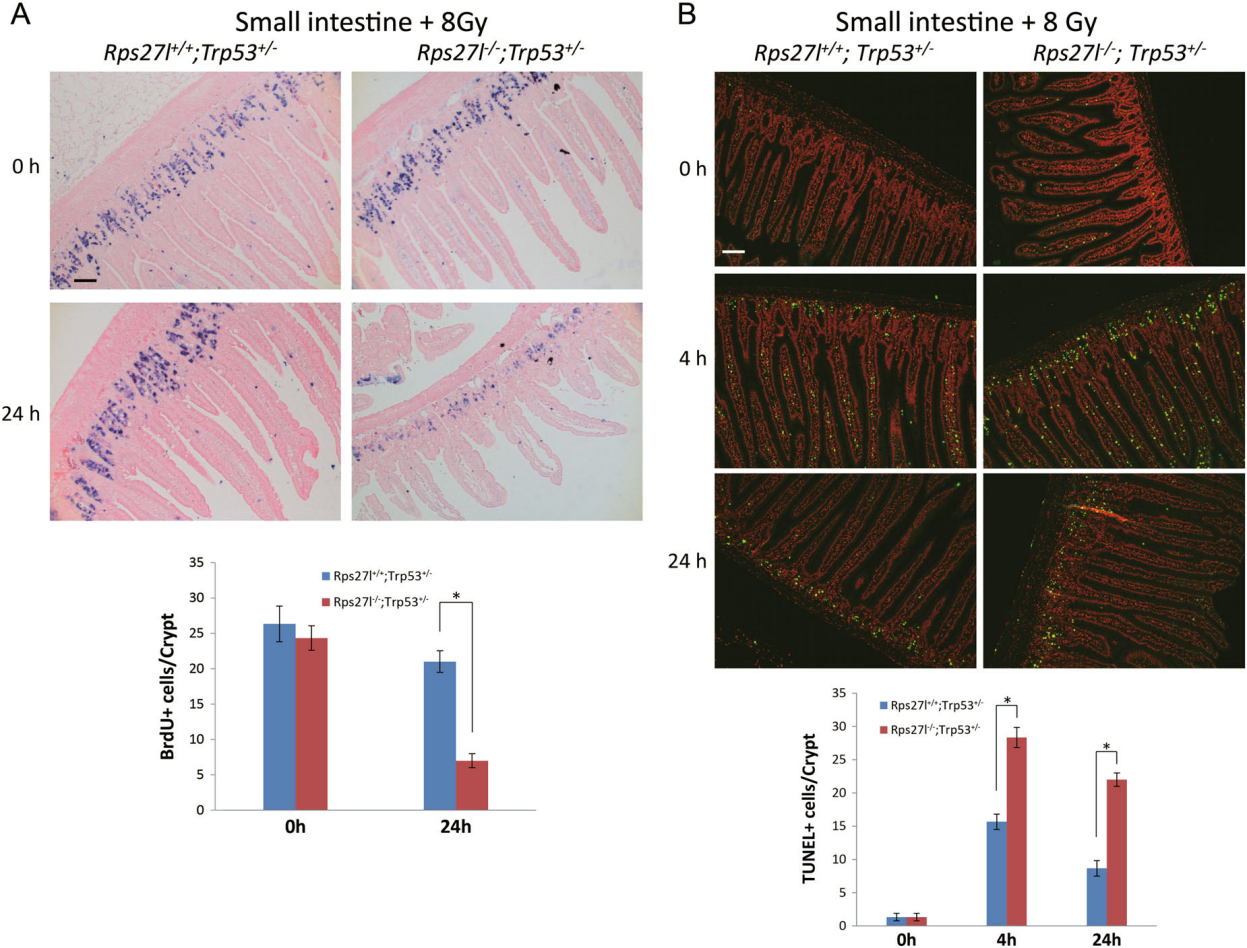
*Rps27l* inactivation reduces proliferation and enhances apoptosis upon radiation. Inactivation of *Rps27l* inhibits proliferation and induces apoptosis upon radiation in small intestine. BrdU was i.p. injected into WT littermates and Rps27l mutants 2 h before mice being sacrificed. Small intestine tissues were harvested, followed by BrdU staining **a** and TUNEL assay **b**. Positive staining cells of each crypt were counted from at least three randomly selected microscopic fields from three independent mice (lower panels). *p < 0.05. Scale bars represent 100 µm

We next determined the cause(s) of mouse death by 8 Gy radiation with focus on cell proliferation by BrdU incorporation and apoptosis by TUNEL and cleaved caspase-3 staining assays in radiation-sensitive organs, including small intestine, thymus and spleen. Compared to *Rps27l*^+/+^; *Trp53*^+/*−*^ mice, *Rps27l*^*−*/*−*^; *Trp53*^+/*−*^ mice showed significantly reduced proliferation in small intestine (Fig. 2a) and spleen (Figure S1A) 24 h post radiation. Remarkably increased apoptosis was readily observed 4 or 24 h post 8 Gy of radiation using TUNEL assay (Fig. 2b), while only a moderate increase of cleaved caspase-3 positively stained cells was seen 4 h post 15 Gy of radiation in small intestine (Figure S1B) due to the sensitivity of different assays and tissues. Indeed, we observed more cleaved caspase-3 positively stained cells 4 or 24 h post 8 Gy of radiation in thymus and spleen (Figure S1C and D). We further employed clonogenic assay to analyze the radiosensitivity of immortalized MEFs, and found that *Rps27l*^*−/−*^*; Trp53*^*+/−*^ MEFs were more sensitive to radiation than *Rps27l*^*+/+*^*; Trp53*^*+/−*^ MEFs (Figure S1E). Collectively, *Rps27l* inactivation confers radiosensitivity in *Trp53*^*+/−*^ mice with reduced proliferation and enhanced apoptosis in radiation-sensitive organs.

### *Rps27l* inactivation increases p53 activity in *Trp53*^*+/−*^ mice

**Fig. 3:**
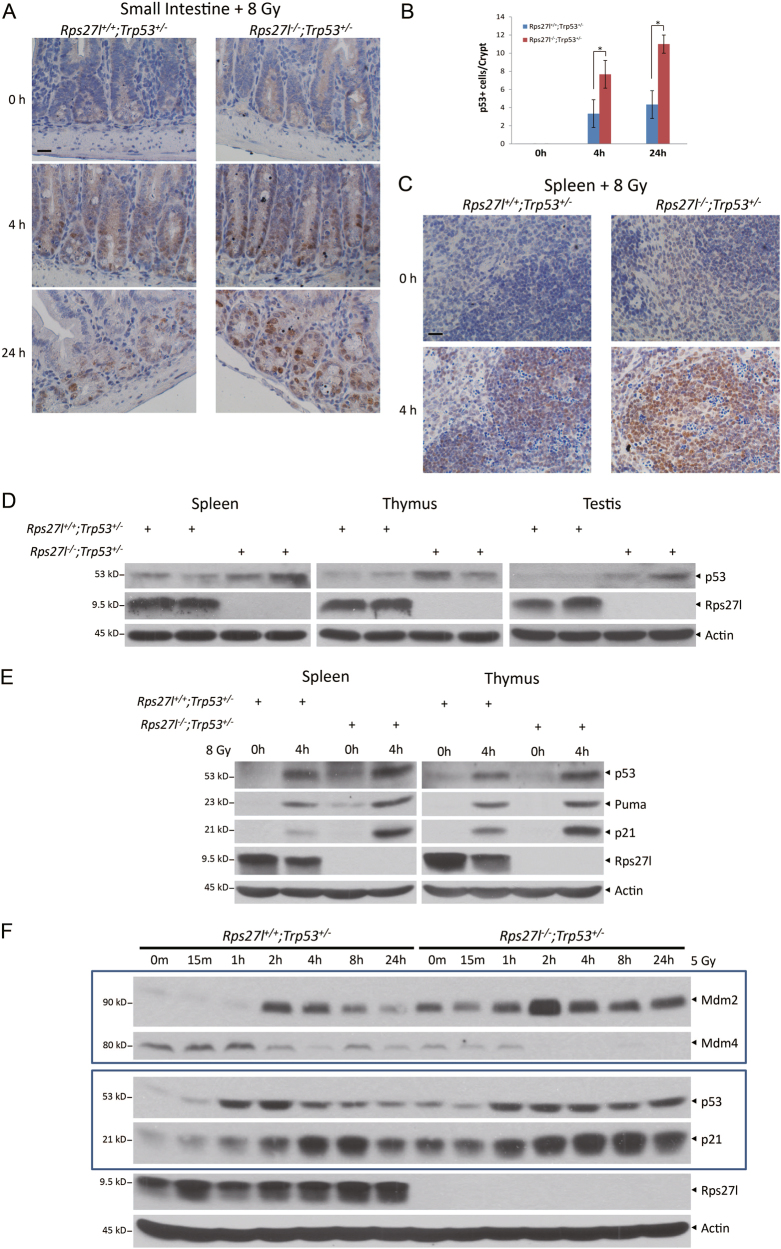
*Rps27l* inactivation increases the levels of p53 and its targets in *Trp53*^*+/*−^ mice. **a**–**c** Inactivation of Rps27l induces the levels of p53 in small intestine (**a**, **b**) and spleen (**c**) of *Trp53*^*+/−*^ mice upon radiation. Mice with indicated genotypes were irradiated at 8 Gy, and then small intestine (**a**, **b**) and spleen (**c**) were collected at indicated time points, followed by IHC staining with p53 antibody and photograph. Positive staining cells of each crypt were counted from at least three randomly selected microscopic fields (**b**). *p < 0.05. Scale bars represent 20 µm. **d**, **e** Inactivation of *Rps27l* induces p53 levels in multiple organs of *Trp53*^*+/−*^ mice with or without radiation. The indicated organs from two individual male mice with indicated genotypes were homogenized and subjected to IB with indicated antibodies. **f** Inactivation of *Rps27l* induces the levels of p53 and its targets in *Trp53*^*+/−*^ MEFs upon radiation. MEFs with indicated genotypes were irradiated at 5 Gy. Cells were then harvested at indicated time points post irradiation, followed by IB with indicated antibodies

To investigate potential molecular mechanisms by which *Rps27l* inactivation confers radiosensitivity in *Trp53*^*+/−*^ mice, we first focused on p53, since (1) tumor suppressor p53 is a key regulator of cellular response to radiation^20^, and (2) our recent study showed that *Rps27l* disruption causes a moderate increase of p53 and its targets in multiple organs, including spleen, bone marrow and fetal liver as well as MEFs^19^. Indeed, as compared to *Rps27l*^*+/+*^*; Trp53*^*+/−*^ littermates, *Rps27l*^*−/−*^*; Trp53*^*+/−*^ mice showed higher p53 levels under unstressed condition in small intestine (Fig. 3a, b), spleen (Fig. 3c), thymus (Figure S2A), and bone marrow (Figure S2B) by immunostaining. Higher p53 levels were also found in spleen, thymus, and testis from two pairs of *Rps27l*^*−/−*^*; Trp53*^*+/−*^ mice by immunoblotting (Fig. 3d). Upon ionizing radiation, increased levels of p53 and its two well-known targets p21 and Puma were found in spleen and thymus from *Rps27l*^*−/−*^*; Trp53*^*+/−*^ mice, as compared to those from *Rps27l*^*+/+*^*; Trp53*^*+/−*^ mice (Fig. 3e).

Our previous study revealed that under physiological *Trp53*^*+/+*^ background, *Rps27l* inactivation triggers ribosomal stress to stabilize Mdm2, which degrades Mdm4 to reduce Mdm2-Mdm4 E3 ligase towards p53, leading to p53 accumulation and activation^19^. We extended this observation to *Trp53*^*+/−*^ MEFs and found that compared to wild-type cells, the levels of Mdm2 or Mdm4 in *Rps27l-*null cells were increased or decreased, respectively, upon radiation with consequent increased duration of p53 stabilization and p21 levels (Fig. 3f). Thus, *Rps27l* deletion under *Trp53*^*+/−*^ background alters the levels of Mdm2 and Mdm4, leading to activation of p53 and its downstream targets in multiple radiation-sensitive organs, followed by reduced proliferation and enhanced apoptosis, and eventually radiosensitization.

### *Rps27l* inactivation impairs DNA damage response to radiation

**Fig. 4:**
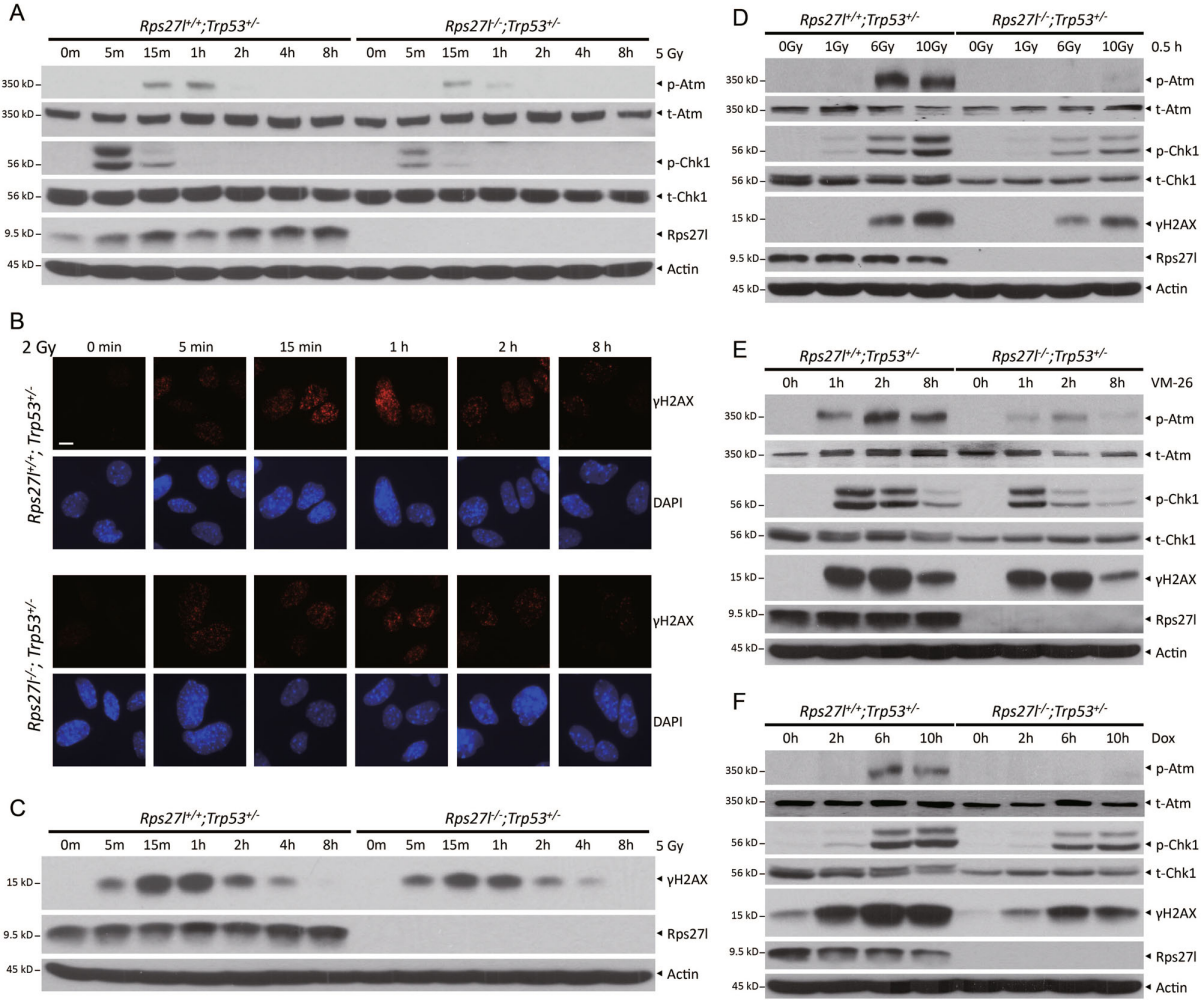
*Rps27l* inactivation impairs the activation of Atm and DNA damage response. **a**–**c** Inactivation of *Rps27l* impairs DNA damage response in *Trp53*^*+/−*^ MEFs upon radiation. MEFs with indicated genotypes were irradiated for indicated periods of time, followed by IB with indicated antibodies (**a**, **c**) or immunofluorescent staining (**b**). **d**–**f** Inactivation of *Rps27l* impairs DNA damage response in *Trp53*^*+/−*^ MEFs upon different doses of radiation (**d**) or upon treatment with DNA damage agents: 5 µM VM-26 (**e**) or 1 µM doxorubincin (Dox) (**f**) for indicated time periods, followed by IB with indicated antibodies

We next determined if radiosensitization by *Rps27l* inactivation under *Trp53*^*+/−*^ background can also be attributable to altered DNA damage response to radiation. We first compared activation of Atm and Chk1 between paired *Rps27l*^*+/+*^*; Trp53*^*+/−*^ and *Rps27l*^*−/−*^*; Trp53*^*+/−*^ MEFs at multiple time points post radiation, and found reduced activation, as reflected by reduced levels of Atm and Chk1 phosphorylation in *Rps27l*^*−/−*^ MEFs (Fig. 4a). Consistently, by immunofluorescent staining and immunoblotting, we detected the lower levels of γH2AX, a hallmark of DNA damage, in *Rps27l*^*−/−*^*; Trp53*^*+/−*^ MEFs at multiple time points post radiation (Fig. 4b, c). Moreover, impaired activation of Atm and Chk1 was also seen in *Rps27l*^*−/−*^*; Trp53*^*+/−*^ MEFs after exposure to different doses of radiation (Fig. 4d), or to different chemotherapeutic agents, such as VM-26 (Fig. 4e) and doxorubincin (Fig. 4f).

**Fig. 5:**
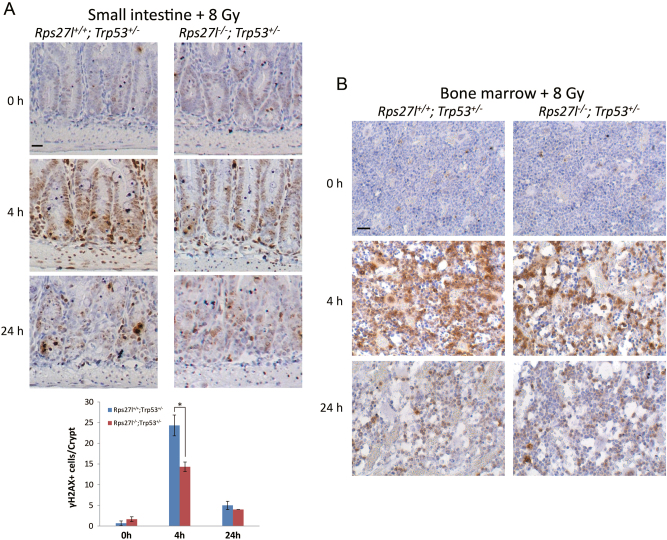
*Rps27l* Inactivation of impairs radiation-induced DNA damage response in small intestine and bone marrow. **a**, **b** Mice with indicated genotypes were irradiated at 8 Gy, small intestine (**a**) and bone marrow (**b**) were harvested at indicated time points, followed by IHC staining with γH2AX antibody. Positive staining cells of each crypt were counted from at least 3 randomly selected microscopic fields (**a**, lower panel). *p < 0.05. Scale bars represent 20 µm

We extended γH2AX immunofluorescent staining to mouse organs and found that the number of γH2AX positively stained cells was significantly reduced in the crypts of small intestine (Fig. 5a) and bone marrow (Fig. 5b) of *Rps27l*^*−/−*^*; Trp53*^*+/−*^ mice upon radiation exposure. Thus, *Rps27l* inactivation impairs Atm activation and subsequent DNA damage response to genotoxic agents.

### *Rps27l* inactivation impairs Atm activation via increased Mdm2 binding of Nbs1

**Fig. 6:**
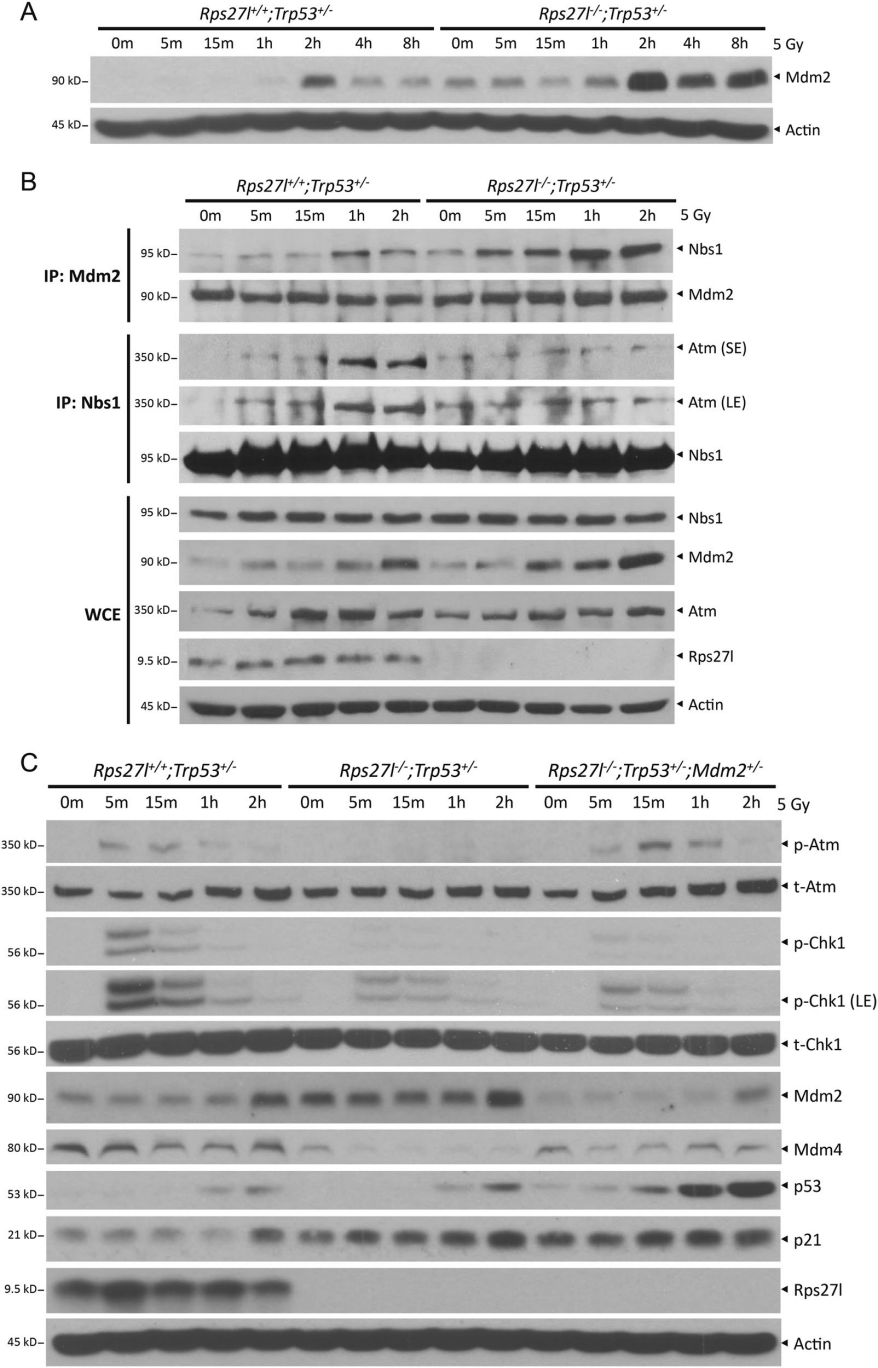
*Rps27l* inactivation impairs the activation of Atm via increased binding of Mdm2 with Nbs1. **a** Inactivation of *Rps27l* induces Mdm2 levels in *Trp53*^*+/−*^ MEFs. MEFs with indicated genotypes were left untreated or irradiated at 5 Gy. Cells were then harvested at indicated time points post irradiation, followed by IB with indicated antibodies. **b** Inactivation of *Rps27l* increases the binding of Mdm2 with Nbs1 and decreases the binding of Nbs1 with Atm in *Trp53*^*+/−*^ MEFs. MEFs with indicated genotypes were left untreated or irradiated at 5 Gy. Cells were then harvested at indicated time points post irradiation, and subjected to immunoprecipitation with Mdm2 or Nbs1 antibody, followed by IB with indicated antibodies. **c** Reduction of Mdm2 rescues the impairment of DNA damage response induced by *Rps27l* inactivation in *Trp53*^*+/−*^ MEFs. MEFs with indicated genotypes were left untreated or irradiated at 5 Gy. Cells were then harvested at indicated time points post irradiation, followed by IB with indicated antibodies. SE shorter exposure, LE longer exposure, WCE whole-cell extract

MDM2 was reported to negatively affect genomic stability independent of p53 by directly binding to Nbs1 of the Mre11-Rad50-Nbs1 (MRN) complex, thus impairing ATM activation and subsequent DNA damage response^21–23^. We found that compared to *Rps27l*^*+/+*^*; Trp53*^*+/−*^ MEFs, *Rps27l*^*−/−*^*; Trp53*^*+/−*^ MEFs had significantly higher Mdm2 levels under unstressed condition or at multiple time points post radiation (Fig. 3f and Fig. 6a). We then determined whether elevated Mdm2 binds more Nbs1 to abrogate MRN-induced Atm activation. Indeed, in two pull-down assays, we detected more Nbs1 in Mdm2 immunoprecipitates and lesser Atm in Nbs1 immunoprecipitates in *Rps27l*^*−/−*^*; Trp53*^*+/−*^ MEFs than in *Rps27l*^*+/+*^*; Trp53*^*+/−*^MEFs at multiple time points post radiation (Fig. 6b), suggesting increased Mdm2–Nbs1 binding and decreased Nbs1–Atm binding upon *Rps27l* inactivation. A causal role of Mdm2 in the process was demonstrated, since simultaneous deletion of one allele of *Mdm2* restored the impaired DNA damage response by *Rps27l* inactivation, as reflected by the recovery of the phosphorylated levels of Atm in *Rps27l*^*−/−*^*; Trp53*^*+/−*^*;Mdm2*^*+/−*^ MEFs (Fig. 6c). Interestingly, phosphorylated Chk1 levels were not significantly higher in *Rps27l*^*−/−*^*; Trp53*^*+/−*^*;Mdm2*^*+/−*^ MEFs than in *Rps27l*^*−/−*^*; Trp53*^*+/−*^*;Mdm2*^*+/+*^ MEFs, suggesting that Mdm2 deletion does not rescue defective ATR activation (Fig. 6c).

Finally, we attempted to determine whether increased Mdm2 plays a causal role in conferring radiosensitization in vivo by generating mice with background of *Rps27l*^*−/−*^*; Trp53*^*+/−*^*;Mdm2*^*+/−*^. However, heterozygous deletion of Mdm2 increased the basal level of p53 (Fig. 6c), and these mice were short-lived with 80% of them died within 6 weeks after birth (Figure S3), further suggesting a feedback loop of the Mdm2/Mdm4–p53 axis to precisely regulate the p53 levels. Taken together, our results demonstrate that *Rps27l* inactivation causes increased Mdm2–Nbs1 binding with subsequent decreased Nbs1–Atm binding to block radiation-induced Atm activation, leading to impaired DNA damage response and enhanced sensitivity to radiation.

## Discussion

Several ribosomal proteins, including RPL37^24^, RPS7^25^, RPL26^26^, and RPS26^27^, were reported to play a role in the maintenance of genomic stability through multiple p53-dependent mechanisms by stabilizing p53 via Mdm2 inhibition, directly increasing p53 translation, or promoting p53 transactivation of its downstream targets in response to DNA damage insults^15,28^. Our recent study using a knockout mouse model demonstrated that *Rps27l* is required for genomic stability under a *Trp53*^*+/−*^ background and *Rps27l*^*−/−*^*; Trp53*^*+/−*^ mice eventually develop spontaneous lymphoma after selective deletion of remaining wild-type *Trp53* allele^19^. In this study, we attempted to examine potential role of Rps27l in radiation-induced tumorigenesis and unexpectedly found that inactivation of *Rps27l* greatly sensitizes *Trp53*^*+/−*^ mice to ionizing radiation at both low and high doses (Fig. 1). A careful examination of radiosensitive organs such as spleen, thymus and small intestine of *Rps27l*^*−/−*^*; Trp53*^*+/−*^ mice revealed reduced proliferation and greatly enhanced apoptosis (Fig. 2), indicating that enhanced radiosensitivity is likely due to the failure of these organs.

As a master regulator of cellular responses to stress and a guardian of genome, p53 is precisely regulated to a low level mainly by Mdm2, a major E3 ubiquitin ligase for targeted p53 ubiquitylation and degradation^29–31^, and Mdm4, an Mdm2 family member, which forms a heterodimer with Mdm2 to have an optimal E3 ligase activity toward p53^32–34^. Our previous study showed that the levels of p53 are moderately induced due to reduced Mdm2/Mdm4 E3 ligase activity toward p53 upon *Rps27l* inactivation, leading to depletion of hematopoietic stem cells via apoptosis and eventual postnatal death^19^. This death phenotype is fully rescued by simultaneous deletion of one allele of *Trp53*^19^. Here we showed that the p53 levels are slightly higher in few organs and MEFs derived from *Rps27l*^*−/−*^*; Trp53*^*+/−*^ mice than those from *Rps27l*^*+/+*^*; Trp53*^*+/−*^ mice, and can be further induced by DNA damaging agents (Fig. 3) with mechanism again involving Mdm2 accumulation and Mdm4 degradation (Figs. 3, 4 and 6a). Therefore, Rps27l is required to keep p53 levels precisely in check when wild-type p53 is present, regardless with one or both alleles of *Trp53* within a cell. It is worth noting that a slight increase of p53 levels is largely dispensable to the survival of *Rps27l*^*−/−*^*; Trp53*^*+/−*^ mice under unstressed condition, as evidenced by their significant extension of life span^19^. However, further induction of p53 levels by radiation exposure (even with a low dose) significantly accelerates death, making it impossible to evaluate radiation-induced tumorigenesis.

We also made a novel observation that *Rps27l* depletion under *Trp53*^*+/−*^ background greatly reduces DNA damage response to the damaging agents (Figs. 4 and 5). Mechanistically, we found, by using paired MEFs derived from littermate embryos with genotypes of *Rps27l*^*+/+*^*; Trp53*^*+/−*^ vs. *Rps27l*^*−/−*^*; Trp53*^*+/−*^, that *Rps27l* inactivation causes increased binding of Mdm2–Nbs1 and decreased binding of Nbs1–Atm, leading to reduced Atm activation and DNA damage response upon radiation exposure (Fig. 6b). Our results are in consistence with recent reports that MDM2 may negatively regulate genomic stability independent of p53 by directly binding to Nbs1 of the MRN complex to impair MRN complex-mediated ATM activation and subsequent DNA damage response in other model systems^21–23,35^. We also found that Mdm2 indeed plays a causal role in the process, since simultaneous deletion of one *Mdm2* allele to reduce Mdm2 levels rescued defective Atm activation (Fig. 6c). Given that RPS27L binding region on MDM2 (amino acids 151–293) overlaps with MDM2-binding region (amino acids 198–314) to NBS1^21,36^, it is very likely that Rps27l depletion vacates the Nbs1-binding region on Mdm2 to facilitate Mdm2–Nbs1 binding. It would be intriguing to investigate whether other MDM2-binding ribosomal proteins (particularly those reported to maintain genomic stability) with overlapping NBS1-binding region, affect DNA damage response by regulating MDM2–NBS1 binding. Finally, it is worth noting that when Mdm2 dose is reduced by one *Mdm2* allele deletion in *Rps27l*^*−/−*^*; Trp53*^*+/−*^ MEFs, we observed a recovery of Mdm4 levels, as well as increased p53 levels (Fig. 6c). The further increase of p53 appears to be sufficient to cause the death, since the life span is significantly shortened in *Rps27l*^*−/−*^*; Trp53*^*+/−*^*;Mdm2*^*+/−*^ mice (Figure S3). Thus, the components of the Rps27l-Mdm2-Mdm4 axis coordinately cross-talk with each other to precisely regulate p53 levels.

**Fig. 7:**
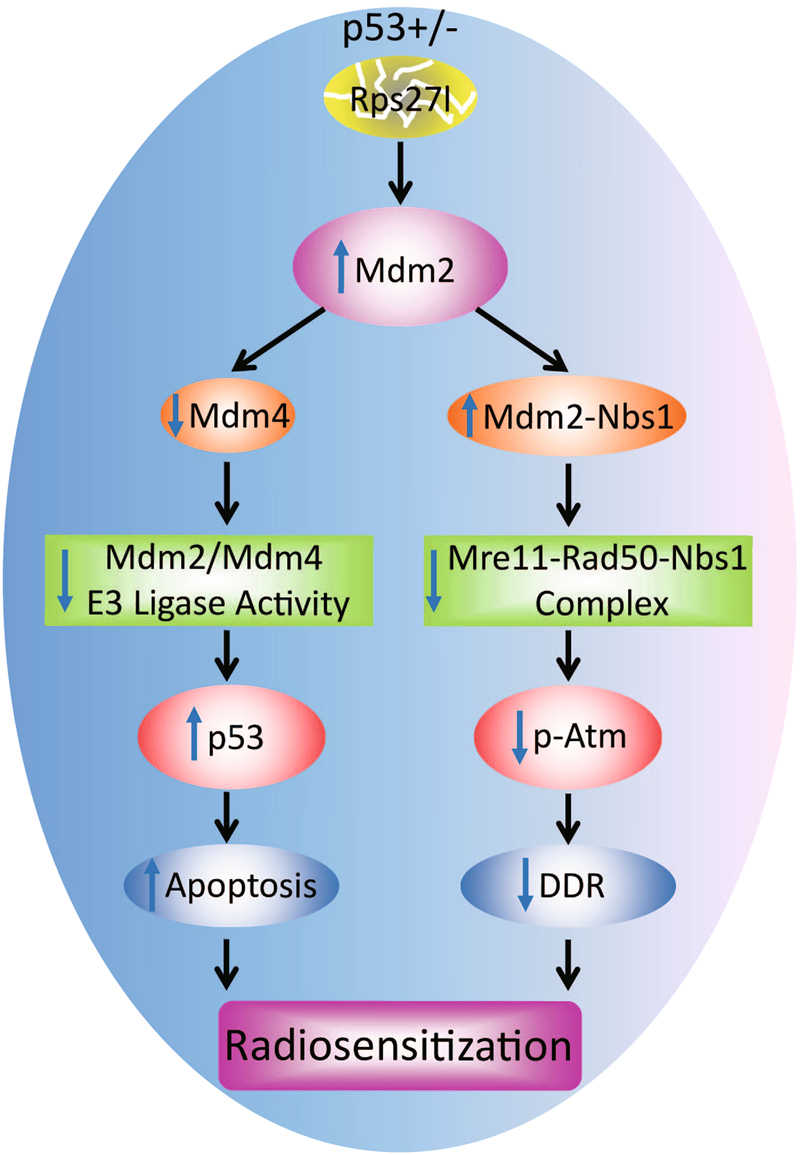
A model for radiosensitivity upon inactivation of ribosomal protein S27l mediated by the Mdm2-p53 and Mdm2–MRN–ATM axes. *Rps27l* inactivation in the *Trp53*^*+/−*^ background triggers Mdm2 accumulation, which on one hand increases p53 by degrading Mdm4, leading to reduced ligase activity toward p53, and on the other hand binds to Nbs1 to reduce its binding and MRN activation of Atm. Increased p53 induces apoptosis, whereas reduced Atm activation impairs DNA damage response, leading to enhanced sensitivity to radiation

In summary, we made here a novel in vivo observation that inactivation of *Rps27l* in p53 heterozygous background significantly sensitizes mouse to radiation by induction of massive apoptosis in multiple radiation-sensitive organs. Two underlying mechanisms were involved. First, ribosomal stress triggered by *Rps27l* inactivation stabilizes Mdm2 levels, leading to the imbalanced Mdm2 vs. Mdm4 levels and reduced E3 ligase activity toward p53^19^. As a result, p53 levels are increased to induce apoptosis, particularly upon radiation exposure. Second, the increased Mdm2 promotes its Nbs1 binding and consequently reduces Nbs1–Atm binding to inhibit Atm activation, leading to reduced DNA damage response (Fig. 7). Although both mechanisms contribute to increased radiosensitivity, the fact that *Rps27l* depletion causes an increased p53 stabilization and activity (Fig. 3f) and a reduced Atm activation (Fig. 4), which would reduce p53 activation, suggests that the Mdm2-Mdm4 axis plays a more important role than the Mdm2–Nbs1–Atm axis in controlling p53 levels and activity under *Rps27l*^*−*/*−*^; *Trp53*^+/*−*^ background. Collectively, our study reveals a physiological relevant condition under which Rps27l regulates the Mdm2-p53 and Mdm2–MRN–ATM axes to maintain DNA damage response and to confer radioprotection. Our study also provides a rationale for enhancing the efficacy of radiation therapy by inactivating RPS27L in human cancers with *TP53*^*+/−*^ status.

## Materials and methods

### Mouse studies

The *Rps27l* gene-trapped mice and *Trp53*-deficient mice were obtained and genotyped as previously described^19^. All procedures were approved by the University of Michigan Committee on Use and Care of Animals. Animal care was provided in accordance with the principles and procedures outlined in the National Research Council Guide for the Care and Use of Laboratory Animals.

### Radiation exposure and clonogenic assay

Mice aged at 8–10 weeks were exposed to different doses of radiation (Philips RT250, Kimtron Medical), followed by survival monitor or being sacrificed at indicated time points. Spontaneously immortalized MEFs were seeded in 60 mm dishes and exposed to different doses of radiation, followed by culture at 37 °C for 7 days. Survival curves were fitted using the linear quadratic equation and the mean inactivation dose was calculated^37,38^.

### Generation and maintenance of MEFs

MEF cells were generated from day E13.5 embryos with indicated genotypes as described^39^, and cultured in DMEM with 15% FBS, 2 mM l-glutamine, 0.1 mM MEM non-essential amino acids at 37 °C in a 5% CO_2_ humidified chamber.

### Western blotting and immunoprecipitation

Cells or tissues were collected, lysed, and subjected to western blotting or immunoprecipitation^19^, using various antibodies as follows: RPS27L polyclonal rabbit antibody was raised and purified as described^17^, p53 (1C12 from Cell Signaling), Mdm2 4B2 and Mdm4 7A8 (gifts from Dr. Jiandong Chen), p21 (556430 from BD Pharmingen), Puma (Cell Signaling), γH2AX (JBW301 from Millipore), pho-ATM (Rockland), ATM (Cell Signaling), pho-Chk1 (Cell Signaling), Chk1 (Santa Cruz), NBS1 (Cell Signaling), rabbit polyclonal Mdm2 Ab for IP raised and purified as described^19^, and β-actin (Sigma).

### Immunohistochemistry

Tissues were fixed in 10% formalin and embedded in paraffin. Sections were cut in 5μm-thick and subjected to immunohistochemical staining^40^. Briefly, following dewaxing, rehydration, and epitope retrieval, serial sections were labeled with p53 (CM5p, Leica Microsystems), cleaved caspase-3 (Cell Signialing), or γH2AX (Millipore), followed by staining with Vectastain ABC kit (Vector Laboratories). Sections were then developed with DAB (Vector Laboratories) and counterstained with haematoxylin. BrdU staining and TUNEL assay were performed using 5-Bromo-2′-deoxy-uridine labeling and detection kit II (Roche) and In Situ Cell Death Detection Kit (Roche), respectively, as manufacturer’s instructions.

### Statistical analysis

Statistical analysis was performed using two-tailed Student’s *t*-test. Data were expressed as mean ± SD. Survival analysis was performed by Kaplan–Meier analysis. Statistical significance was determined as *p* < 0.05.

## Electronic supplementary material

Supplemental figures and figure legends
