# Supplementary material for: Inactivation of ribosomal protein S27-like confers radiosensitivity via the Mdm2-p53 and Mdm2–MRN–ATM axes
Source: Cell Death Dis. 2018 Feb 2;9(2):145. doi: 10.1038/s41419-017-0192-3 (PMC5833388; doi:10.1038/s41419-017-0192-3)

Supplemental Materials

Figure S1. *Rps27l* inactivation reduces proliferation and induces apoptosis in the spleen and thymus of *Trp53*$^{+/-}$ mice upon radiation exposure.

(**A**) *Rps27l* inactivation inhibits proliferation of splenocytes in *Trp53*$^{+/-}$ mice upon radiation. BrdUwas i.p. injected into WT littermates and Rps27l mutants 2 hours before mice being sacrificed. Spleens were harvested, followed by BrdU staining. Scale bar represents 200 μm.

(**B-D**) *Rps27l* inactivation induces apoptosis in small intestine (B), thymuses (C) and spleens (D) from *Trp53*$^{+/-}$ mice upon radiation. Mice with indicated genotypes were sacrificed at indicated time points post irradiation. Tissues were harvested and subjected to IHC staining with cleaved caspase-3 antibody. Scale bar represents 100 μm.

(**E**) Inactivation of *Rps27l* sensitizes *Trp53*$^{+/-}$ MEFs to radiation. Immortalized MEFs were seeded in 60 mm dishes in triplicate and irradiated. The colonies with more than 50 cells were counted after 7 days. Surviving fraction was calculated as the proportion of seeded cells following irradiation to form colonies relative to that of *Rps27l*$^{+/+}$; *Trp53*$^{+/-}$ MEFs (mean ± SEM, n= 3).

Figure S2. *Rps27l* inactivation causes an increase of p53 and its targets upon radiation.

(**A&B**) *Rps27l* inactivation increases p53 staining in the thymus (A) and bone marrow (B) of *Trp53*$^{+/-}$ mice upon radiation exposure. Mice with indicated genotypes were irradiated at 8 Gy, thymuses were harvested or femurs from hind limb were fixed at indicated time points, followed by IHC staining with p53 antibody. Scale bar represents 20 μm.

Figure S3. Heterozygous deletion of Mdm2 shortens the survival of Rps27l-null mice under *Trp53*$^{+/-}$ background.

Kaplan–Meier survival curves of mice with genotype of *Rps27l*$^{-/-}$;*Trp53*$^{+/+}$, *Rps27l*$^{-/-}$;*Trp53*$^{+/-}$ and *Rps27l*$^{-/-}$;*Trp53*$^{+/-}$;*Mdm2*$^{+/-}$. The number in the parenthesis indicates the numbers of mice in each group. Log-rank test, p<0.0001.

Zhao et al. Figure S1

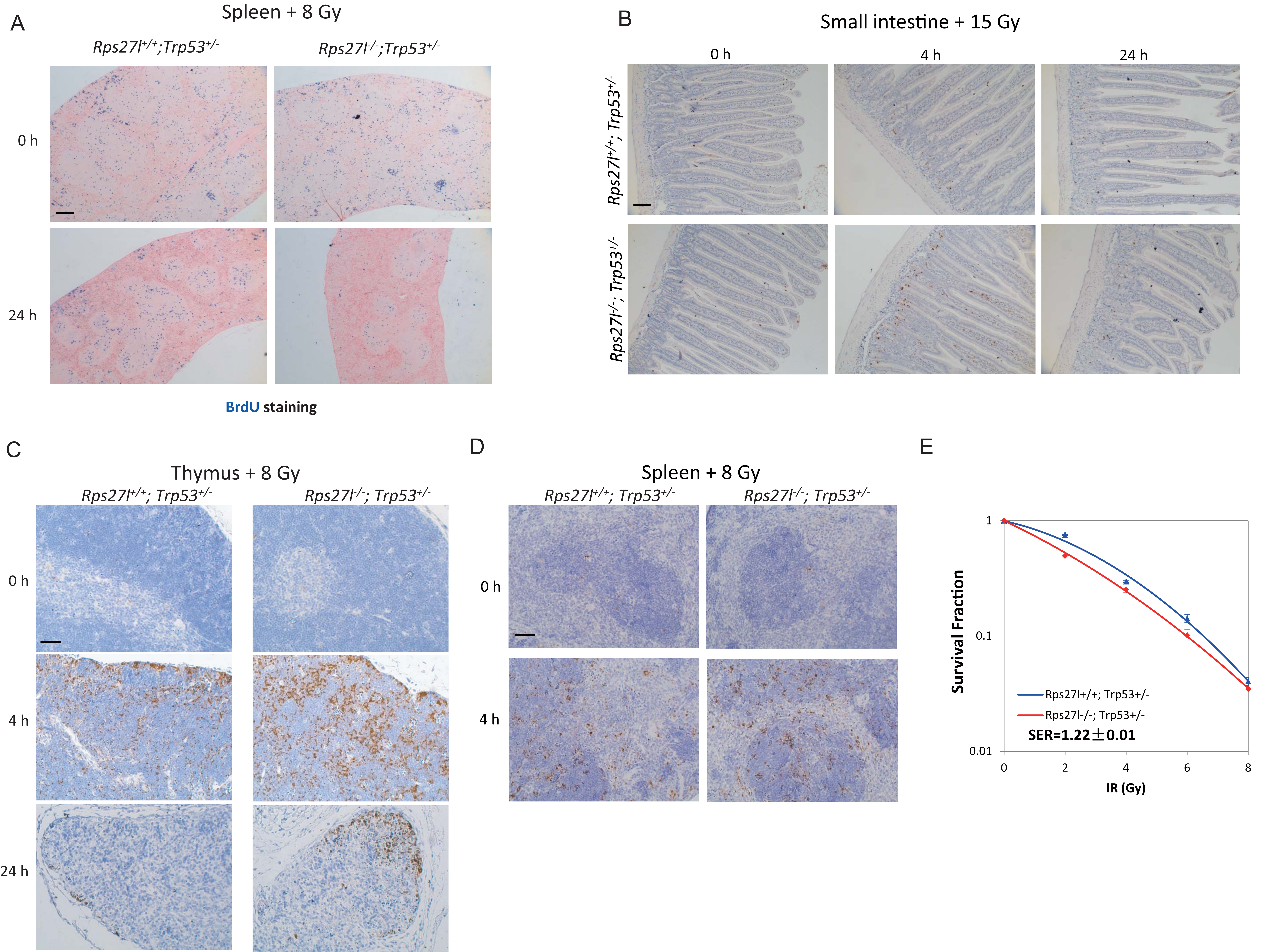

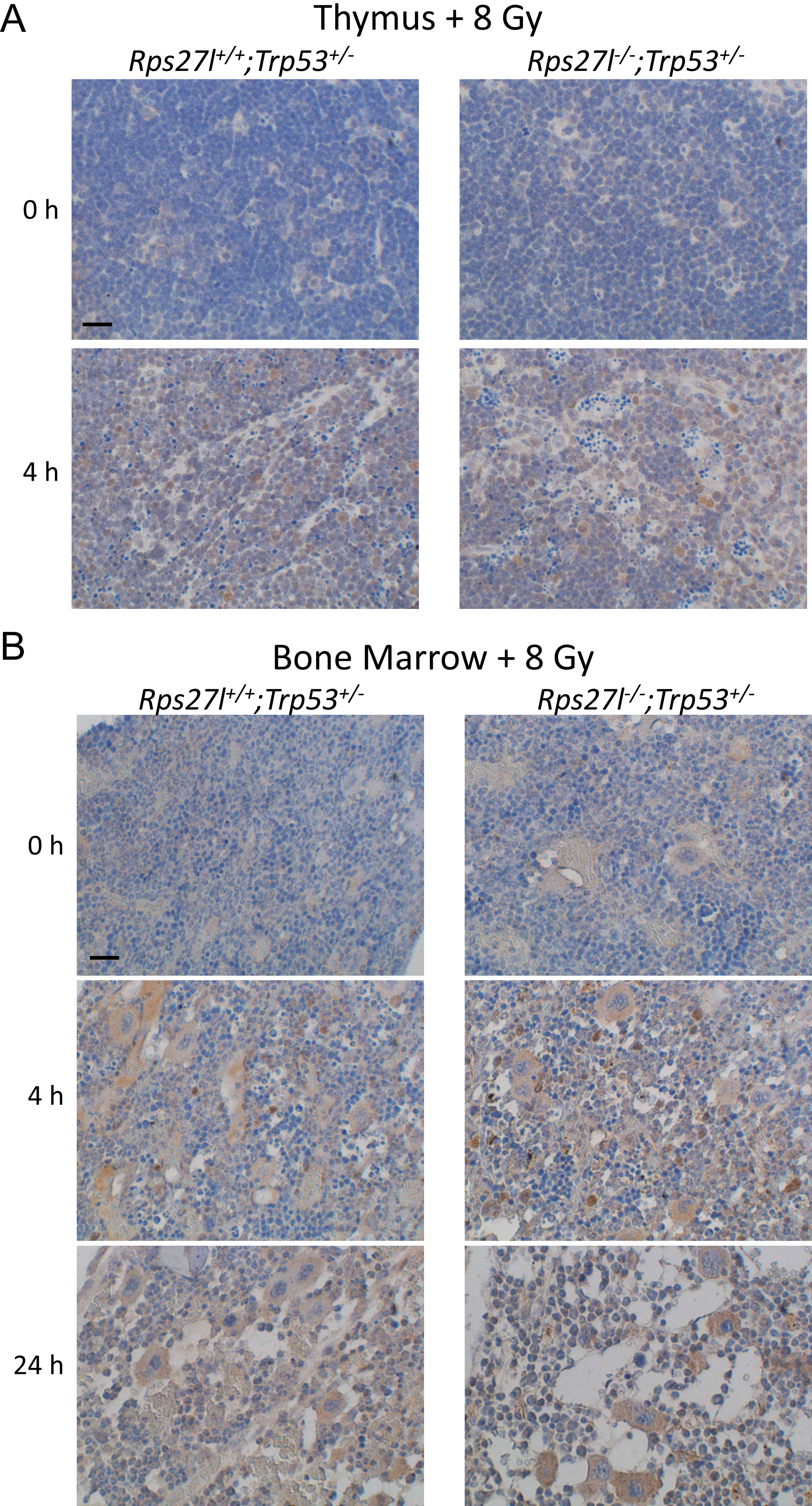
A
Thymus + 8 Gy
Rps27l+/+;Trp53+/-
Rps27l-/-;Trp53+/-
0 h
4 h
B
Bone Marrow + 8 Gy
Rps27l+/+;Trp53+/-
Rps27l-/-;Trp53+/-
0 h
4 h
24 h

Zhao et al. Figure S3

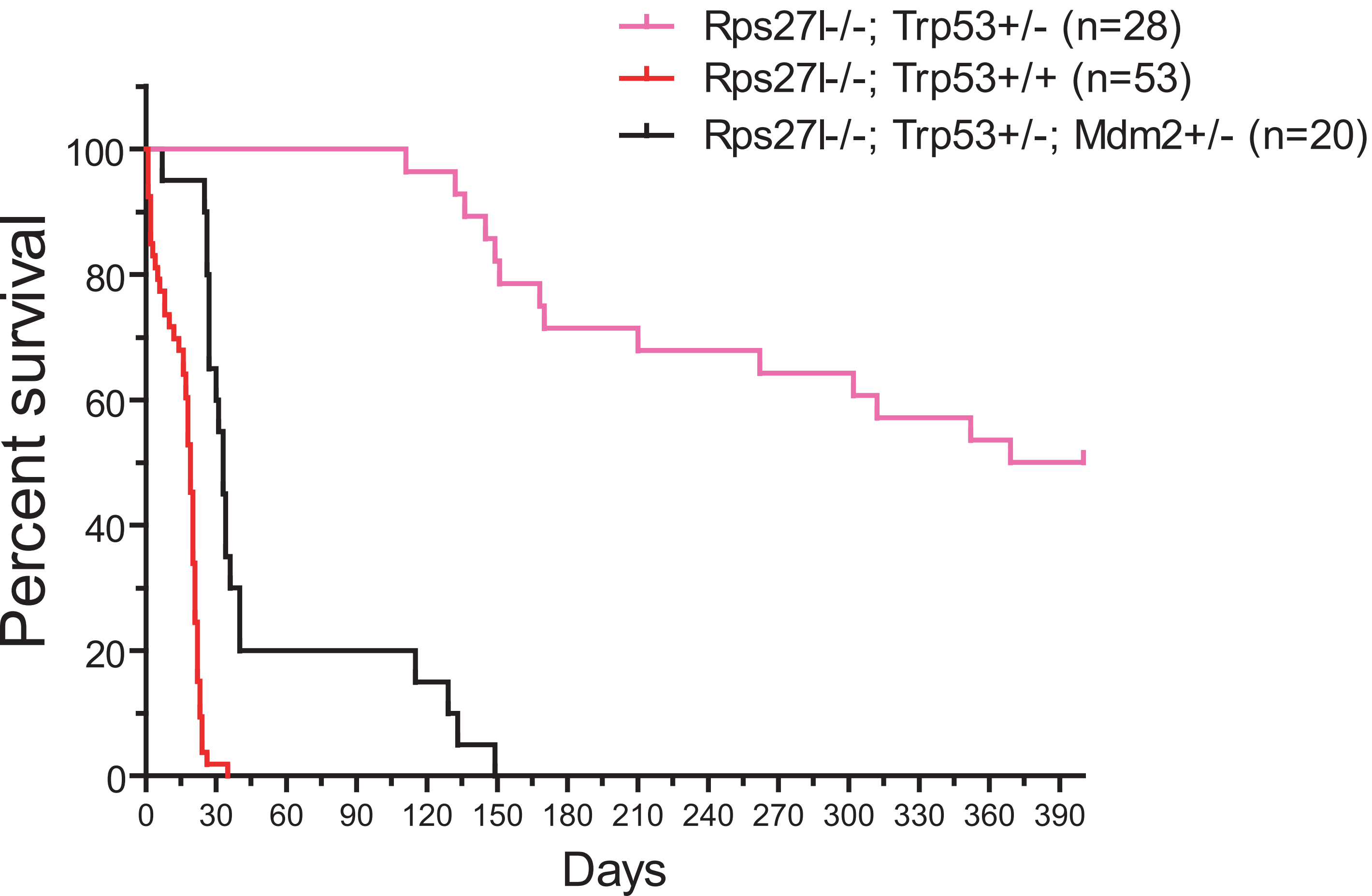

Supplement: Supplementary file 1 — Supplemental figures and figure legends [file 41419_2017_192_MOESM1_ESM.pdf]
